# Interaction between obstructive sleep apnea and short sleep duration on insulin resistance: a large-scale study: OSA, short sleep duration and insulin resistance

**DOI:** 10.1186/s12931-020-01416-x

**Published:** 2020-06-16

**Authors:** Huajun Xu, Chen Liang, Jianyin Zou, Hongliang Yi, Jian Guan, Meizhen Gu, Yanhong Feng, Shankai Yin

**Affiliations:** 1 Department of Otolaryngology Head and Neck Surgery & Center of Sleep Medicine, Shanghai Jiao Tong University Affiliated Sixth People’s Hospital, Yishan Road 600, Shanghai, 200233 China; 2 Otolaryngological Institute of Shanghai Jiao Tong University, Yishan Road 600, Shanghai, 200233 China; 3 Shanghai Key Laboratory of Sleep Disordered Breathing, Yishan Road 600, Shanghai, 200233 China; 4 Drug clinical trial institution, Shanghai Jiao Tong University Affiliated Sixth People’s Hospital, Yishan Road 600, Shanghai, 200233 China; 5 Department of Otolaryngology-Head & Neck Surgery, Shanghai Children’s Hospital, Shanghai Jiao Tong University, Huding Road 355, Shanghai, 200062 China; 6 Department of Ultrasound, The First Affiliated Hospital of Jinzhou Medical University, No. 2, 5 Duan, Renmin Street, Guta District, Jinzhou, 121000 Liaoning Province China

**Keywords:** Obstructive sleep apnea, Insulin resistance, Sleep duration

## Abstract

**Objectives:**

Both short sleep duration and obstructive sleep apnea (OSA) seem to be associated with insulin resistance. We aimed to explore whether short sleep duration modifies the relationship between OSA and insulin resistance.

**Methods:**

Participants were consecutively enrolled from our sleep center during the period from 2007 to 2017. The index of homeostasis model assessment insulin resistance (HOMA-IR) was calculated from insulin and glucose. Sleep duration was determined by standard polysomnography. The associations between sleep duration and insulin resistance were estimated by logistic regression analyses.

**Results:**

A total of 5447 participants (4507 OSA and 940 primary snorers) were included in the study. OSA was independently correlated with insulin resistance after adjusting for all potential confounders (OR, 1.319; 95% CI, 1.088–1.599), but not short sleep duration. In stratified analysis by sleep duration, compared with primary snorers, in the OSA group only extremely short sleep duration (< 5 h) was significantly associated with insulin resistance after adjusting for all covariates (OR, 2.229; 95% CI, 1.283–3.874). Rapid eye movement predominant OSA was significantly associated with insulin resistance (OR = 1.355, 95% CI: 1.019–1.802) after adjustment for confounding factors including age, sex and body mass index.

**Conclusions:**

OSA, but not short sleep duration, was independently associated with insulin resistance. It is worth noting that OSA combined with extremely short sleep duration showed a greater detrimental effect than OSA itself with regard to insulin resistance.

## Introduction

Obstructive sleep apnea (OSA), which is the most common form of sleep-disordered breathing, affects about 34% of men and 17% of women in middle to older age groups [1]. A recent meta-analysis estimated that 936 million adults worldwide aged 30–69 years have mild to severe OSA. In parallel, the prevalence of insufficient sleep duration due to bedtime restriction, short-term sleep fragmentation, and sleep deprivation is also increasing in modern societies. Both OSA and short sleep duration have attracted a great deal of attention, not only as they are common globally, but also as they are considered to be independent risk factors for cognitive, metabolic, and cardiovascular problems [2–6].

One study showed that short sleep duration is independently associated with hypertension in OSA patients [7]. However, another study indicated that OSA, but not short sleep duration, was independently associated with obesity, hypertension, and dyslipidemia [8]. In patients with pre-diabetes, self-reported short sleep duration was associated with adverse measures of glycemia [9]. However, after considering the effect of OSA, sleep duration was not significantly associated with an abnormal fasting glucose level [10]. Considering these inconsistent results, studies on OSA should account for sleep duration and vice versa.

Several cross-sectional and longitudinal studies showed that OSA was independently correlated with insulin resistance [11, 12]. Insulin resistance may also mediate the relationships of common cardiovascular risk factors with OSA. Similarly, short sleep duration is also correlated with lower insulin sensitivity [13] and increased insulin resistance, even after adjusting for adiposity [14]. Recently, insulin resistance, characterized by a decreased cellular response to insulin, was shown to have a significant impact on various adverse clinical outcomes, including hypertension, diabetes mellitus, nonalcoholic fatty liver disease, cardiovascular diseases, and even kidney diseases [15]. However, few recent studies have explored whether OSA and short sleep duration are independently correlated with insulin resistance. One study showed that subjects in the shortest sleep duration group (< 5 h) had 14.5% higher fasting insulin level and 16.3% higher homeostasis model assessment (HOMA)-β; these associations disappeared after adjustment for body mass index (BMI) and sleep apnea [16]. However, this previous study did not assess OSA by standard polysomnography (PSG) and included no objective measure of sleep duration. In addition, these associations have not been explored in Han Chinese populations.

We used a large-scale hospital-based cross-sectional dataset, including 5447 participants, to examine 1) whether objectively measured short sleep duration and OSA are independently associated with insulin resistance, and 2) whether the presence of OSA modulates the association between sleep duration and insulin resistance.

## Methods

This cross-sectional study enrolled consecutive patients referred to the sleep center of Shanghai Jiao Tong University Affiliated Sixth People’s Hospital. The study procedure was approved by the Ethics Committee of Shanghai Jiao Tong University Affiliated Sixth People’s Hospital in accordance with the Declaration of Helsinki (Trial registration number: ChiCTR1900025714). Informed consent was obtained from all subjects.

### Participants

All potential participants completed a comprehensive questionnaire regarding their general health status, including smoking and alcohol use (never, past, or current), and medication use. Smoking were defined as current smokers or subjects who smoked cigarettes before. Non-smoking were defined as subjects who had never smoked cigarettes. The exclusion criteria were as follows: 1) aged < 18 years; 2) previous treatment for OSA; 3) absence of full-lead PSG data; 4) chronic diseases, such as hepatic, pulmonary, or cardiac disease; 5) comorbid sleep disorders, such as insomnia, central sleep apnea (CSA), upper airway resistance syndrome (UARS), restless leg syndrome (RLS), and narcolepsy; 6) current use of anxiolytics, hypnotics, antidepressants, or other antipsychotics; and 7) had anti-diabetes drugs.

### Clinical and biochemical measurements

Height, weight, waist circumference (WC), neck circumference (NC), and hip circumference (HC) were measured as described previously, without shoes and while wearing lightweight clothing [17]. BMI was calculated as weight in kilograms divided by height in meters squared. After a rest period of about 15 min, the systolic blood pressure (SBP) and diastolic blood pressure (DBP) were measured using a standard mercury sphygmomanometer. All of the above indices were measured twice and mean values of the two measurements were calculated.

Fasting venous blood was collected from each participant at 07:00. The lipid profile [total cholesterol (TC), triglycerides (TG), high-density lipoprotein cholesterol (HDL-C), low-density lipoprotein cholesterol (LDL-C), apolipoprotein A-I (ApoA-I), apolipoprotein B (ApoB), apolipoprotein E (ApoE), and lipoprotein(a) (Lp(a))] was measured in the clinical laboratory of our hospital. Fasting serum glucose was determined using an H-7600 autoanalyzer (Hitachi, Tokyo, Japan), and serum insulin was determined by an immunoradiological method. The index of homeostasis model assessment insulin resistance (HOMA-IR) was calculated as fasting insulin (μU/mL) × fasting glucose (mmol/L)/22.5 [18]. Insulin resistance was defined as HOMA-IR ≥ 2.5 [19]. Hypertension was defined as diagnosis provided by the referring physician, and by the use of cardiovascular medications that were specifically used as antihypertensive medications. Diabetes was defined as the use of antidiabetic medication and fasting plasma glucose ≥7.0 mmol/L. Dyslipidemia was defined as use of lipid-lowering medication or serum TC level > 200 mg/dL, LDL-C level > 130 mg/dL, HDL-C level < 39.8 mg/dL, or TG level > 150 mg/dL, according to the diagnostic criteria of the National Cholesterol Education Program Adult Treatment Panel III (NCEP ATP III) [20].

### Sleep evaluation

All participants completed the Chinese version of the Epworth Sleepiness Scale (ESS) to evaluate daytime sleepiness; the total ESS scores range from 0 to 24 [21]. An ESS score > 10 was defined as excessive daytime sleepiness (EDS) [22].

All participants underwent overnight standard PSG in a sound-attenuated, light- and temperature-controlled room in our sleep center. Participants were asked to arrive at least 2 h before the sleep study to familiarize themselves with the sleep environment. Bedtime was adjusted according to participants’ habitual sleep time, with the recording time ranging from 21:00–22:00 to 06:00–07:00.

Objective sleep data were obtained via standard PSG (Alice 4 or 5; Respironics, Pittsburgh, PA). Polysomnographic monitoring was performed in accordance with the 2007 guidelines of the American Academy of Sleep Medicine (AASM) [23]. The PSG was equipped with six electroencephalography (EEG) channels (F3–F4, C3–C4, and O1–O2), submental electromyography (EMG) channels, bilateral electrooculogram (EOG) channels, and electrocardiography (ECG) channels, as well as a nasal pressure transducer, oronasal thermistor, piezo belts, and snoring sound and pulse oximetry sensors. Apnea was defined as an at-least 90% reduction in airflow for at least 10 s; hypopnea was defined as an at-least 50% reduction in airflow for at least 10 s, accompanied by an at-least 3% reduction in oxygen saturation or arousal. The indices derived from full-night PSG included total sleep time (TST), percentage of each sleep stage (N1, N2, N3, and REM), sleep efficiency, apnea hypopnea index (AHI) [23], lowest oxygen saturation (LSpO_2_), oxygen desaturation index (ODI), mean oxygen saturation, and microarousal index (MAI). Sleep duration was defined according to the TST, as described previously [ [7, 24]]. The sleep duration was divided into five categories: > 8, 7–8, 6–7, 5–6, and < 5 h. OSA was classified into four classes: < 5, 5 ≤ AHI < 15, 15 ≤ AHI < 30, and AHI ≥ 30. AHI_REM_ and AHI_NREM_ were calculated as the number of apnea and hypopnea events per hour of REM and NREM sleep, respectively. REM predominant OSA was defined as AHI_REM_ ≥ 5 and AHI_NREM_ < 5. A skilled technician manual checked the data according to the AASM 2007 guidelines [23].

### Statistical analysis

All statistical analyses were performed using SPSS software (version 22.0; IBM SPSS Statistics, IBM Corp., Armonk, NY). Normally distributed continuous data are shown as means ± SD and were analyzed using one-way analysis of variance (ANOVA) and the independent-samples *t* test. Skewed data are presented as interquartile range (IQR) and were compared by the Mann–Whitney U test. Categorical data are presented as percentages and were analyzed using the χ^2^ test. *P*-values for linear trends across groups were calculated using the polynomial linear trend test.

Independent associations of OSA and sleep duration with insulin resistance were assessed by logistic regression. The covariates adjusted for in our study included age, gender, BMI, smoking, alcohol consumption, hypertension, hyperlipidemia, time in bed, time in sleep stage N3 (%), ESS, AHI (or TST), waist hip ratio, diabetes mellitus, ApoA-I, ApoB, ApoE, and Lp(a). The adjusted odds ratio (ORs) and 95% confidence intervals (CIs) for insulin resistance were calculated according to OSA status and sleep duration. Finally, the joint effects of OSA and sleep duration were examined using primary snorers with a sleep duration of 7–8 h as the reference group. In all analyses, *P* < 0.05 was taken to indicate statistical significance.

## Results

A total of 5447 participants (4507 OSA and 940 primary snorers) were included in this study; their demographic and sleep characteristics by OSA severity are presented in Table 1. Patients with OSA were predominantly male, more obese, older, and had poorer metabolic and sleep profiles. Table 2 presents the demographic characteristics and sleep parameters by sleep duration. Patients with shorter sleep duration were older, less obese, had lower AHI, and lower likelihood of insulin resistance (Table 2).

**Table 1:** Demographic and sleep characteristics across OSA severity categories

Characteristics	Total OSA (*n* = 4507)	Mild OSA (*n* = 878)	Moderate OSA (*n* = 891)	Severe OSA (*n* = 2738)	P
**Demographic and clinical characteristics**					
Men, n (%)	3819 (84.73)	663 (75.51)	715 (80.25)	2441 (89.15)	<.0001^*^
Age (years)	43.52 ± 11.67	42.19 ± 12.14	44.71 ± 12.41	43.56 ± 11.22	<.0001^*^
Neck circumference (cm)	39.91 ± 3.48	38.19 ± 3.28	39.02 ± 3.28	40.74 ± 3.34	<.0001^*^
Waist circumference (cm)	97.29 ± 10.40	92.10 ± 10.25	94.72 ± 9.48	99.81 ± 9.88	<.0001^*^
Hip circumference (cm)	102.10 ± 7.81	99.44 ± 7.52	100.65 ± 7.51	103.50 ± 7.69	<.0001^*^
Waist hip ratio	0.95 ± 0.18	0.92 ± 0.07	0.95 ± 0.31	0.97 ± 0.18	<.0001^*^
BMI (kg/m^2^)	27.25 ± 3.85	25.53 ± 3.35	26.37 ± 3.60	28.08 ± 3.84	<.0001^*^
Hypertension, n (%)	1676 (37.19)	212 (24.15)	310 (34.79)	1154 (42.15)	<.0001^*^
SBP (mmHg)	127.7 ± 15.64	124.53 ± 14.85	126.03 ± 16.31	129.28 ± 15.46	<.0001^*^
DBP (mmHg)	82.18 ± 11.73	79.19 ± 11.23	80.21 ± 11.39	83.76 ± 11.72	<.0001^*^
Diabetes mellitus, n (%)	409 (9.07)	54 (6.15)	68 (7.63)	287 (10.48)	0.0001^*^
Hyperlipidemia, n (%)	1396 (25.63)	165 (18.79)	228 (25.59)	885 (32.32)	<.0001^*^
Smoking, n (%)	1956 (43.40)	291 (33.14)	344 (38.61)	1321 (48.25)	<.0001^*^
Alcohol consumption, n (%)	533 (11.83)	61 (6.95)	78 (8.75)	394 (14.39)	<.0001^*^
Fasting glucose (mmol/l)	5.60 ± 1.29	5.38 ± 1.28	5.50 ± 1.25	5.70 ± 1.30	<.0001^*^
Fasting insulin (μU/ml)	11.70 (7.86,17.23)	9.38 (6.29,14.21)	10.48 (7.28,15.26)	12.91 (8.88,18.83)	<.0001^*^
HOMA-IR	2.81 (1.81,4.36)	2.12 (1.41,3.41)	2.46 (1.66,3.72)	3.14 (2.08,4.84)	<.0001^*^
Cholesterol (mmol/l)	4.87 ± 1.00	4.74 ± 0.99	4.78 ± 0.89	4.93 ± 1.03	<.0001^*^
Triglyceride (mmol/l)	2.16 ± 1.88	1.80 ± 1.63	1.97 ± 1.43	2.34 ± 2.05	<.0001^*^
High density lipoprotein (mmol/l)	1.05 ± 0.25	1.10 ± 0.27	1.07 ± 0.25	1.03 ± 0.24	<.0001^*^
Low density lipoprotein (mmol/l)	3.05 ± 0.83	2.99 ± 0.84	3.01 ± 0.80	3.09 ± 0.83	0.0018^*^
ApolipoproteinA-1(g/l)	1.07 ± 0.19	1.08 ± 0.20	1.08 ± 0.21	1.06 ± 0.18	0.0254^*^
Apolipoprotein-B(g/l)	0.88 ± 0.20	0.84 ± 0.19	0.86 ± 0.19	0.90 ± 0.21	<.0001^*^
Apolipoprotein-E (mg/dl)	4.81 ± 2.28	4.37 ± 1.64	4.69 ± 2.90	4.99 ± 2.21	<.0001^*^
Lipoprotein-α (mg/dl)	12.22 ± 14.22	13.90 ± 16.49	12.80 ± 14.89	11.50 ± 13.14	<.0001^*^
ESS	9.12 ± 5.87	7.03 ± 5.15	7.36 ± 5.21	10.36 ± 5.95	<.0001^*^
ESS > 10, n (%)	1773 (39.34)	217 (24.72)	239 (26.82)	1317 (48.10)	<.0001^*^
**Polysomnography**					
Total sleep duration, min	398.1 ± 76.20	385.35 ± 78.45	389.58 ± 77.67	404.96 ± 74.18	<.0001^*^
Total time in bed, min	491.3 ± 94.89	491.36 ± 91.61	491.62 ± 93.78	491.24 ± 96.31	0.9944
Sleep efficiency (%)	0.82 ± 0.15	0.79 ± 0.15	0.80 ± 0.16	0.84 ± 0.14	<.0001^*^
S1(% TST)	18.38 ± 14.23	16.74 ± 12.84	17.64 ± 13.61	19.14 ± 14.79	<.0001^*^
S2(% TST)	49.08 ± 19.80	47.66 ± 18.93	47.97 ± 19.58	49.90 ± 20.11	0.0024^*^
S3(% TST)	12.81 ± 10.88	15.40 ± 10.93	14.04 ± 10.39	11.57 ± 10.83	<.0001^*^
REM(% TST)	10.43 ± 6.57	10.90 ± 6.89	10.57 ± 6.62	10.23 ± 6.44	0.0246^*^
AHI (events/h)	41.80 ± 25.31	9.55 ± 2.94	22.03 ± 4.26	58.58 ± 17.41	<.0001^*^
LSpO_2_,%	74.95 ± 13.46	85.94 ± 9.13	81.36 ± 8.96	69.34 ± 12.72	<.0001^*^
ODI	42.11 ± 26.83	10.72 ± 9.04	23.12 ± 9.59	58.35 ± 20.77	<.0001^*^
Mean SaO_2_	93.33 ± 3.56	95.67 ± 1.51	95.01 ± 1.68	92.03 ± 3.86	<.0001^*^
MAI	31.52 ± 22.24	20.47 ± 18.53	23.44 ± 16.68	37.70 ± 22.70	<.0001^*^

Acronyms: *OSA* Obstructive sleep apnea, *BMI* Body mass index, *HOMA-IR* Homeostasis model assessment for insulin resistance, *SBP* Systolic blood pressure, *DBP* Diastolic blood pressure, *ESS* Epworth Sleepiness Scale, *AHI* Apnea–hypopnea index, *TST* Total sleep time, *REM* Rapid eye movement, *LSpO*_*2*_ Lowest oxygen saturation, *ODI* Oxygen desaturation index, *SaO*_*2*_ Oxygen saturation

The data are presented as means and standard deviation (SD) and categorical data as the number (percentage)

Differences in the baseline characteristics among the four groups were examined using the polynomial linear trend test for continuous variables and the linear-by-linear association test for dichotomous variables

* *p* indicates a significant difference

**Table 2:** Demographic and sleep characteristics of all participants stratified by total sleep duration categories

Characteristics	> 8 h (*n* = 538)	7-8 h (*n* = 1757)	6-7 h (*n* = 1710)	5-6 h (*n* = 841)	≤5 h (*n* = 601)	P
**Demographic and clinical characteristics**						
Men,n(%)	441 (81.97)	1457 (82.93)	1387 (81.11)	676 (80.38)	471 (78.37)	0.1297
Age (years)	42.88 ± 11.53	42.18 ± 11.50	42.24 ± 11.59	43.02 ± 12.03	44.11 ± 13.05	0.0045^*^
Neck circumference (cm)	39.91 ± 3.92	39.47 ± 3.52	39.35 ± 3.77	39.18 ± 3.70	38.78 ± 3.65	<.0001^*^
Waist circumference (cm)	97.78 ± 12.15	96.09 ± 10.50	95.31 ± 11.16	94.86 ± 11.48	93.27 ± 11.20	<.0001^*^
Hip circumference (cm)	102.63 ± 8.28	101.57 ± 7.77	101.26 ± 8.05	100.77 ± 8.01	99.83 ± 7.51	<.0001^*^
Waist hip ration	0.95 ± 0.07	0.95 ± 0.22	0.94 ± 0.22	0.94 ± 0.07	0.93 ± 0.07	0.2733
BMI (kg/m^2^)	27.44 ± 4.37	26.89 ± 3.91	26.69 ± 4.04	26.42 ± 3.88	25.99 ± 3.80	<.0001^*^
Hypertension, n (%)	154 (28.62)	459 (26.12)	431 (25.20)	199 (23.66)	153 (25.46)	0.0005^*^
SBP (mmHg)	127.84 ± 15.31	127.36 ± 15.86	125.46 ± 15.38	126.24 ± 15.76	125.57 ± 15.91	0.3267
DBP (mmHg)	82.90 ± 11.81	81.96 ± 11.68	80.77 ± 11.66	81.07 ± 11.57	80.09 ± 11.01	<.0001^*^
Diabetes mellitus, n (%)	54 (10.04)	158 (8.99)	132 (7.72)	61 (7.25)	42 (6.99)	0.1603
Hyperlipidemia,n (%)	421 (78.25)	1410 (80.25)	1304 (76.26)	641 (76.22)	451 (75.04)	0.0160^*^
Smoking, n (%)	250 (46.47)	765 (43.54)	697 (40.76)	314 (37.34)	178 (29.62)	<.0001^*^
Alcohol consumption, n(%)	69 (12.83)	181 (10.30)	203 (11.87)	80 (9.51)	43 (7.15)	0.0059^*^
Fasting glucose (mmol/l)	5.67 ± 1.38	5.57 ± 1.30	5.47 ± 1.22	5.48 ± 1.22	5.40 ± 1.15	0.0006^*^
Fasting insulin (μU/ml)	10.93 (7.74,16.52)	11.20 (7.59,16.22)	10.89 (7.13,16.26)	11.11 (7.04,16.90)	10.47 (6.96,15.61)	0.1768
HOMA-IR	2.57 (1.78,4.19)	2.65 (1.73,4.17)	2.56 (1.60,4.03)	2.63 (1.59,4.14)	2.50 (1.55,3.94)	0.0313^*^
Cholesterol (mmol/l)	4.85 ± 0.94	4.80 ± 1.02	4.75 ± 0.96	4.81 ± 1.02	4.81 ± 1.12	0.2433
Triglyceride (mmol/l)	2.17 ± 2.03	2.11 ± 1.88	2.01 ± 1.80	2.03 ± 1.83	1.91 ± 1.46	0.0633
High density lipoprotein (mmol/l)	1.04 ± 0.23	1.04 ± 0.24	1.07 ± 0.25	1.10 ± 0.26	1.12 ± 0.30	<.0001^*^
Low density lipoprotein (mmol/l)	2.95 ± 0.76	2.97 ± 0.82	2.99 ± 0.82	3.03 ± 0.83	3.07 ± 0.97	0.0471^*^
ApolipoproteinA-1(g/l)	1.06 ± 0.18	1.05 ± 0.18	1.08 ± 0.20	1.09 ± 0.21	1.10 ± 0.21	<.0001^*^
Apolipoprotein-B (g/l)	0.88 ± 0.21	0.88 ± 0.21	0.85 ± 0.20	0.85 ± 0.20	0.85 ± 0.22	<.0001^*^
Apolipoprotein-E (mg/dl)	4.76 ± 2.14	4.72 ± 2.56	4.61 ± 1.96	4.75 ± 2.10	4.61 ± 1.70	0.3730
Lipoprotein-α (mg/dl)	13.78 ± 15.85	12.59 ± 15.06	11.99 ± 13.93	12.52 ± 14.71	13.50 ± 16.72	0.0839
ESS	9.01 ± 5.70	8.97 ± 5.96	8.55 ± 5.83	7.97 ± 5.76	7.73 ± 5.74	<.0001^*^
ESS > 10, n(%)	203 (37.73)	670 (38.13)	629 (36.78)	259 (30.80)	179 (29.78)	<.0001^*^
**Polysomnography**						
Total sleep duration, min	501.31 ± 17.48	447.95 ± 16.64	394.27 ± 17.51	333.97 ± 16.91	235.34 ± 58.19	<.0001^*^
Total time in bed, min	547.02 ± 71.76	512.54 ± 89.05	480.98 ± 83.65	472.24 ± 99.31	445.39 ± 124.62	<.0001^*^
Sleep efficiency,%	0.92 ± 0.07	0.89 ± 0.09	0.84 ± 0.11	0.73 ± 0.12	0.56 ± 0.18	<.0001^*^
S1(% TST)	16.08 ± 12.44	16.50 ± 13.11	17.04 ± 13.70	20.47 ± 13.99	22.70 ± 16.36	<.0001^*^
S2(% TST)	53.30 ± 16.15	50.09 ± 19.99	47.61 ± 20.75	48.46 ± 17.41	46.74 ± 19.14	<.0001^*^
S3(% TST)	15.24 ± 11.36	13.42 ± 10.52	12.70 ± 10.64	13.69 ± 10.80	13.17 ± 12.04	0.0001^*^
REM(% TST)	12.23 ± 6.49	10.89 ± 6.56	10.60 ± 6.76	10.15 ± 6.35	9.38 ± 7.42	<.0001^*^
AHI (events/h)	40.93 ± 27.23	37.10 ± 27.51	34.97 ± 27.60	31.08 ± 27.10	28.66 ± 26.16	<.0001^*^
Lowest-SpO_2_,%	74.05 ± 13.93	75.96 ± 14.47	77.81 ± 13.97	79.87 ± 14.58	81.65 ± 15.00	<.0001^*^
ODI	41.40 ± 29.11	37.32 ± 28.60	35.17 ± 28.66	32.76 ± 28.96	31.33 ± 28.31	<.0001^*^
Mean SaO_2_	93.11 ± 3.93	93.62 ± 3.55	93.83 ± 3.68	94.34 ± 2.94	94.60 ± 2.71	<.0001^*^
OSA, n (%)	466 (86.62)	1493 (84.97)	1411 (82.51)	680 (80.86)	457 (76.04)	<.0001^*^
Mild OSA, n (%)	57 (10.59)	262 (14.90)	284 (16.61)	163 (19.38)	112 (18.64)	<.0001^*^
Moderate OSA, n (%)	80 (14.87)	268 (15.25)	273 (15.96)	164 (19.50)	106 (17.64)	0.0514
Severe OSA, n (%)	329 (61.15)	963 (54.81)	854 (49.94)	353 (41.97)	239 (39.77)	<.0001^*^
MAI	31.44 ± 21.41	28.78 ± 21.37	29.24 ± 22.57	28.86 ± 22.19	27.70 ± 20.03	0.0552

Acronyms: *OSA* Obstructive sleep apnea, *BMI* Body mass index, *HOMA-IR* Homeostasis model assessment for insulin resistance, *SBP* Systolic blood pressure, *DBP* Diastolic blood pressure, *ESS* Epworth Sleepiness Scale, *AHI* Apnea–hypopnea index, *TST* Total sleep time, *REM* Rapid eye movement, *LSpO*_*2*_ Lowest oxygen saturation, *ODI* Oxygen desaturation index, *SaO*_*2*_ Oxygen saturation

The data are presented as means and standard deviation (SD) and categorical data as the number (percentage)

Differences in the baseline characteristics among the five groups were examined using the polynomial linear trend test for continuous variables and the linear-by-linear association test for dichotomous variables

* *p* indicates a significant difference

In whole cohort, compared with primary snorers (non-OSA), OSA was independently correlated with insulin resistance after adjusting for all potential confounders (OR, 1.319; 95% CI, 1.088–1.599) (Table 3). Taking a sleep duration of 7–8 h (optimal sleep time) as the reference, 5–6 h and < 5 h of sleep were associated with an increased risk of insulin resistance (OR, 1.127; 95% CI, 0.921–1.379; and OR, 1.167; 95% CI, 0.927–1.417, respectively) after adjusting for all potential confounders, but not significantly (Table 3).

**Table 3:** adjusted ORs and 95% CIs for the association of insulin resistance and OSA (primary snoring as reference) or sleep duration (sleep time:7-8 h as reference)

predictors	n	OR (95% CI)				
		Model 1	Model 2	Model 3	Model 4	Model 5
**OSA**						
Primary snoring	940	Reference	Reference	Reference	Reference	Reference
OSA	4507	1.593 (1.336–1.900)^*^	1.450 (1.210–1.737) ^*^	1.467 (1.224–1.758) ^*^	1.375 (1.143–1.654) ^*^	1.319 (1.088–1.599) ^*^
**Sleep duration**						
> 8 h	538	0.792 (0.635–0.987) ^*^	0.807 (0.645–1.010) ^*^	0.769 (0.613–0.965) ^*^	0.746 (0.592–0.940) ^*^	0.758 (0.597–0.936) ^*^
7-8 h	1757	Reference	Reference	Reference	Reference	Reference
6-7 h	1710	0.897 (0.771–1.042)	0.936 (0.802–1.091)	0.954 (0.817–1.114)	0.948 (0.810–1.109)	0.972 (0.826–1.144)
5–6	841	1.005 (0.835–1.209)	1.070 (0.885–1.294)	1.122 (0.926–1.359)	1.105 (0.909–1.344)	1.127 (0.921–1.379)
≤ 5 h	601	1.039 (0.843–1.281)	1.085 (0.873–1.348)	1.158 (0.930–1.442)	1.137 (0.909–1.421)	1.167 (0.927–1.417)
P for linear trend		0.1809	0.0716	0.0079	0.0107	0.0076

Model 1 was adjusted for age, sex and BMI; model 2 was adjusted for variables included in model 1 and smoking, alcohol use, hypertension, hyperlipidemia, time in bed, S3, ESS; model 3 was adjusted for variables included in model 2 and TST (or AHI); model 4 was adjusted for variables included in model 3 and waist hip ratio; model 5 was adjusted for variables in model 4 and additional diabetes mellitus, apolipoprotein A-1, apolipoprotein-B, apolipoprotein-E and lipoprotein-α

Acronyms: *OSA* Obstructive sleep apnea, *OR* Odds ratio, *CI* Confidence interval, *BMI* Body mass index, *TST* Total sleep time, *ESS* Epworth Sleepiness Scale, *AHI* Apnea-hypopnea index

* *p* indicates a significant difference

In stratified analysis by sleep duration, we performed logistic regression analyses to evaluate the correlation between insulin resistance and OSA in each subgroup (in > 8, 7–8, 6–7, 5–6, and < 5 h). Compared with primary snorers (non-OSA), in the OSA group only extremely short sleep duration (< 5 h: OR, 2.229; 95% CI, 1.283–3.874) was significantly associated with insulin resistance after adjusting for all covariates (Table 4).

**Table 4:** Adjusted ORs and 95% CIs for the association of insulin resistance with OSA in stratified analysis by sleep duration

Total sleep duration	n	OR (95% CI)				
		Model 1	Model 2	Model 3	Model 4	Model 5
**> 8 h**						
Primary snoring	72	Reference	Reference	Reference	Reference	Reference
OSA	466	1.622 (0.867–3.034)	1.244 (0.639–2.422)	1.250 (0.640–2.440)	1.306 (0.657–2.593)	1.119 (0.545–2.298)
**7-8 h**						
Primary snoring	264	Reference	Reference	Reference	Reference	Reference
OSA	1493	1.332 (0.968–1.833)	1.251 (0.903–1.734)	1.255 (0.905–1.740)	1.127 (0.806–1.575)	1.036 (0.732–1.468)
**6-7 h**						
Primary snoring	299	Reference	Reference	Reference	Reference	Reference
OSA	1411	1.472 (1.076–2.014) ^*^	1.287 (0.930–1.780)	1.286 (0.929–1.780)	1.244 (0.894–1.731)	1.287 (0.908–1.825)
**5-6 h**						
Primary snoring	161	Reference	Reference	Reference	Reference	Reference
OSA	680	1.755 (1.128–2.731)^*^	1.643 (1.042–2.591) ^*^	1.678 (1.062–2.649) ^*^	1.559 (0.980–2.480)	1.454 (0.905–2.336)
**≤5 h**						
Primary snoring	144	Reference	Reference	Reference	Reference	Reference
OSA	457	2.659 (1.628–4.344) ^*^	2.500 (1.508–4.143) ^*^	2.524 (1.524–4.180) ^*^	2.196 (1.304–3.700) ^*^	2.229 (1.283–3.874) ^*^

Model 1 was adjusted for age, sex and BMI; model 2 was adjusted for variables included in model 1 and smoking, alcohol use, hypertension, hyperlipidemia, time in bed, S3, ESS; model 3 was adjusted for variables included in model 2 and TST (or AHI); model 4 was adjusted for variables included in model 3 and waist hip ratio; model 5 was adjusted for variables in model 4 and additional diabetes mellitus, apolipoprotein A-1, apolipoprotein-B, apolipoprotein-E and lipoprotein-α

Acronyms: *OSA* Obstructive sleep apnea, *OR* Odds ratio, *CI* Confidence interval, *BMI* Body mass index, *TST* Total sleep time, *ESS* Epworth Sleepiness Scale, *AHI* Apnea-hypopnea index

* *p* indicates a significant difference

In stratified analysis by OSA severity, we performed logistic regression analyses to evaluate the correlation between insulin resistance and sleep duration in each subgroup (in mild, moderate and severe OSA). When we use primary snorers (non-OSA) as a reference, no significant differences were found in each OSA group (Table 5).

**Table 5:** Adjusted odd ratios (ORs) and 95% confidence interval (CIs) of insulin resistance associated with total sleep duration in stratified analysis by OSA severity

predictors	n	OR(95% CI)				
		Model 1	Model 2	Model 3	Model 4	Model 5
Primary snoring	940	Reference	Reference	Reference	Reference	Reference
**Mild OSA**						
> 8 h	57	0.942 (0.501–1.774)	0.999 (0.523–1.909)	0.920 (0.438–1.930)	0.934 (0.436–1.999)	0.896 (0.402–1.997)
7-8 h	262	1.208 (0.882–1.655)	1.142 (0.828–1.575)	1.055 (0.659–1.688)	1.052 (0.652–1.698)	1.012 (0.613–1.668)
6-7 h	284	0.953 (0.696–1.304)	0.917 (0.665–1.264)	0.845 (0.530–1.352)	0.821 (0.511–1.320)	0.856 (0.521–1.405)
5–6	163	1.163 (0.794–1.705)	1.155 (0.783–1.705)	1.069 (0.639–1.788)	1.054 (0.621–1.788)	1.017 (0.581–1.779)
≤ 5 h	112	1.903 (1.213–2.986) ^*^	1.735 (1.104–2.784) ^*^	1.621 (0.913–2.875)	1.582 (0.876–2.856)	1.693 (0.921–3.112)
**Moderate OSA**						
> 8 h	80	1.150 (0.690–1.920)	1.102 (0.656–1.850)	1.114 (0.494–2.515)	1.164 (0.509–2.664)	1.156 (0.490–2.729)
7-8 h	268	1.325 (0.966–1.816)	1.253 (0.910–1.726)	1.268 (0.620–2.592)	1.270 (0.612–2.635)	1.170 (0.548–2.497)
6-7 h	273	1.300 (0.953–1.773)	1.268 (0.922–1.743)	1.282 (0.626–2.627)	1.343 (0.649–2.777)	1.399 (0.658–2.974)
5–6	164	1.735 (1.183–2.544) ^*^	1.736 (1.176–2.563)	1.755 (0.831–3.707)	1.889 (0.883–4.042)	1.961 (0.893–4.308)
≤ 5 h	106	1.808 (1.154–2.832) ^*^	1.781 (1.128–2.813) ^*^	1.801 (0.817–3.970)	1.824 (0.816–4.076)	2.039 (0.889–4.678)
**Severe OSA**						
> 8 h	329	1.535 (1.131–2.084) ^*^	1.249 (0.912–1.711)	0.510 (0.330–0.788)	0.486 (0.312–0.757)	0.503 (0.318–0.795)
7-8 h	963	1.944 (1.551–2.436) ^*^	1.668 (1.318–2.110) ^*^	0.699 (0.481–1.015)	0.698 (0.478–1.020)	0.718 (0.486–1.063)
6-7 h	854	1.847 (1.464–2.331) ^*^	1.654 (1.298–2.109) ^*^	0.689 (0.471–1.007)	0.683 (0.465–1.004)	0.709 (0.476–1.057)
5–6	353	2.200 (1.637–2.957) ^*^	1.978 (1.459–2.683) ^*^	0.833 (0.548–1.267)	0.787 (0.514–1.205)	0.818 (0.528–1.269)
≤ 5 h	239	2.141 (1.531–2.994)^*^	1.970 (1.389–2.793) ^*^	0.845 (0.539–1.326)	0.823 (0.521–1.300)	0.813 (0.507–1.302)

Model 1 was adjusted for age, sex and BMI; model 2 was adjusted for variables included in model 1 and smoking, alcohol use, hypertension, hyperlipidemia, time in bed, S3, ESS; model 3 was adjusted for variables included in model 2 and TST (or AHI); model 4 was adjusted for variables included in model 3 and waist hip ratio; model 5 was adjusted for variables in model 4 and additional diabetes mellitus, apolipoprotein A-1, apolipoprotein-B, apolipoprotein-E and lipoprotein-α

Acronyms: *OSA* Obstructive sleep apnea, *OR* Odds ratio, *CI* Confidence interval, *BMI* Body mass index, *ESS* Epworth Sleepiness Scale, *AHI* Apnea-hypopnea index

* *p* indicates a significant difference

Besides, we performed a subgroup analysis by sex (male, female), age (< 60 years, ≥60 years) and BMI (< 28 kg/m^2^, ≥28 kg/m^2^), only sleep duration was independently correlated with insulin resistance in younger patients with sleep time < 5 h (OR, 1.35; 95% CI, 1.00–1.83) (Table 6).

**Table 6:** Adjusted odd ratios (ORs) and 95% confidence intervals (CIs) of insulin resistance associated with total sleep duration in different subgroups

predictors	Sex		Age		BMI	
	Men (*n* = 4432)	Women (*n* = 1015)	< 60 y (*n* = 4866)	≥60 y (*n* = 581)	< 28 kg/m^2^ (*n* = 3653)	≥28 kg/m^2^ (*n* = 1794)
Primary snoring	Reference	Reference	Reference	Reference	Reference	Reference
> 8 h	0.683 (0.491–0.949) ^*^	1.087 (0.543–2.177)	0.760 (0.558–1.036)	0.975 (0.336–2.858)	0.743 (0.521–1.061)	0.644 (0.352–1.179)
7-8 h	0.875 (0.677–1.132)	1.549 (0.936–2.566)	0.936 (0.740–1.184)	1.951 (0.793–4.795)	0.957 (0.739–1.240)	0.825 (0.484–1.408)
6-7 h	0.842 (0.651–1.089)	1.439 (0.872–2.377)	0.899 (0.711–1.137)	1.855 (0.753–4.571)	0.916 (0.707–1.186)	0.787 (0.461–1.345)
5-6 h	1.100 (0.826–1.465)	1.454 (0.817–2.589)	1.145 (0.877–1.493)	2.004 (0.781–5.140)	1.141 (0.855–1.524)	1.024 (0.566–1.852)
≤5 h	1.235 (0.897–1.699)	1.752 (0.933–3.288)	1.351 (1.000–1.826)^*^	1.985 (0.753–5.231)	1.223 (0.886–1.689)	1.440 (0.742–2.798)
P for linear trend	0.0018^*^	0.4108	0.0096^*^	0.8965	0.0495^*^	0.0187^*^

Model was adjusted for age, sex, BMI, smoking, alcohol use, hypertension, hyperlipidemia, time in bed, S3, ESS, AHI

Acronyms: *OSA* Obstructive sleep apnea, *OR* Odds ratio, *CI* Confidence interval, *BMI* Body mass index, *TST* Total sleep time, *ESS* Epworth Sleepiness Scale, *AHI* Apnea-hypopnea index

* *p* indicates a significant difference

In order to explore the effect of interaction between OSA and sleep duration on insulin resistance, multiplicative interaction was evaluated with logistic regressions. After adjusting for age, sex, BMI, smoking, alcohol use, hypertension, hyperlipidemia, time in bed, S3, ESS and waist hip ratio, an interaction effect of OSA and extremely short sleep duration (< 5 h) on insulin resistance was found (OR, 1.467; 95% CI, 1.018–2.116), taking primary snorers with a sleep time of 7–8 h as the reference group (Table 7). This interaction disappeared after additional adjustment for diabetes mellitus, ApoA-I, ApoB, ApoE, and Lp(a).

**Table 7:** Adjusted odd ratios (ORs) and 95% confidence intervals (CIs) of interaction between OSA and sleep duration on insulin resistance

predictors	n	OR (95% CI)			
		Model 1	Model 2	Model 3	Model 4
> 8 h*Primary snoring	72	0.633 (0.334–1.200)	0.735 (0.382–1.413)	0.622 (0.338–1.299)	0.670 (0.334–1.343)
> 8 h*OSA	466	1.094 (0.769–1.556)	1.034 (0.723–1.479)	0.931 (0.647–1.340)	0.860 (0.590–1.255)
7-8 h*Primary snoring	264	Reference	Reference	Reference	Reference
7-8 h*OSA	1493	1.338 (0.985–1.818)	1.272 (0.932–1.737)	1.176 (0.858–1.613)	1.070 (0.772–1.485)
6-7 h*Primary snoring	299	0.854 (0.577–1.264)	0.969 (0.650–1.445)	0.936 (0.623–1.405)	0.867 (0.569–1.322)
6-7 h*OSA	1411	1.215 (0.893–1.652)	1.190 (0.869–1.629)	1.099 (0.799–1.510)	1.046 (0.752–1.454)
5-6 h*Primary snoring	161	0.782 (0.489–1.250)	0.836 (0.517–1.350)	0.829 (0.510–1.347)	0.836 (0.509–1.371)
5–6*OSA	680	1.428 (1.027–1.987) ^*^	1.442 (1.030–2.021) ^*^	1.314 (0.933–1.850)	1.224 (0.858–1.744)
≤5 h*Primary snoring	144	0.628 (0.380–1.040)	0.647 (0.387–1.081)	0.638 (0.379–1.072)	0.604 (0.349–1.043)
≤5 h*OSA	457	1.622(1.143–2.303) ^*^	1.615(1.127–2.315) ^*^	1.467(1.018–2.116) ^*^	1.384(0.949–2.020)

Model 1 was adjusted for age, sex and BMI; model 2 was adjusted for variables included in model 1 and smoking, alcohol use, hypertension, hyperlipidemia, time in bed, S3, ESS; model 3 was adjusted for variables included in model 3 and waist hip ratio; model 4 was adjusted for variables in model 4 and additional diabetes mellitus, apolipoprotein A-1, apolipoprotein-B, apolipoprotein-E and lipoprotein-α

Acronyms: *OSA* Obstructive sleep apnea, *OR* Odds ratio, *CI* Confidence interval, *BMI* Body mass index, *ESS* Epworth Sleepiness Scale, *AHI* Apnea-hypopnea index

* *p* indicates a significant difference

In supplementary Table 1, REM predominant OSA was significantly associated with insulin resistance (OR = 1.355, 95% CI: 1.019–1.802) after adjustment for confounding factors including age, sex and BMI. However, though such an association disappeared after too many confounding factors adjusted, the insulin resistance in REM sleep predominant OSA was more severe when compared with primary snorers.

Besides, in multiple logistic regression models, we found almost near all variables listed were significantly associated with insulin resistance (supplementary Table 2).

## Discussion

In this large-scale cross-sectional study, only OSA was significantly correlated with insulin resistance even after adjustment for covariates, including sleep duration. OSA and sleep time < 5 h had an interaction effect on insulin resistance.

The relationship between OSA and insulin resistance has been well documented. AHI was independently correlated with insulin resistance even after adjusting for covariates [25]. Our previous study showed that intermittent hypoxia was independently associated with hyperglycemia, hyperinsulinemia, and insulin resistance [11]. In the Sleep Heart Health Study (SHHS study), sleep fragmentation, another important characteristic of OSA, was also shown to be associated with insulin resistance [26]. Consistent with the above studies, we also found that OSA was independently correlated with insulin resistance, even after controlling for sleep duration. Potential mechanisms linking OSA and insulin resistance include sympathetic nerve excitability, oxidative stress, inflammation, and hypothalamic–pituitary–adrenal (HPA) axis dysfunction [27].

The conclusions of previous studies exploring the correlation between insulin resistance and sleep duration were inconsistent. One study showed that, in comparison to the reference group with a sleep time of 6–8 h, sleep times of < 6 and > 8 h tended to be correlated with lower rates of insulin resistance [28]. Interestingly, short sleep duration was reported to be correlated with insulin resistance in women but not in men [14]. The mechanism by which insulin resistance induces short sleep duration was suggested to involve activation of the HPA axis, resulting in inhibition of pancreatic function and, finally, reduced glucose tolerance and insulin resistance [29]. Other clinical studies reported contradictory findings. One study indicated that neither short nor long sleep duration was correlated with insulin resistance in older non-diabetic patients [30]. Another study showed that long, but not short, sleep duration was associated with insulin resistance in non-diabetic subjects with or without sleep disorders, such as sleep apnea and insomnia [31]. Elsewhere, a long sleep duration was not significantly correlated with HOMA-IR [14]. These discrepancies among studies may have been due to differences in ethnicity, the characteristics of the enrolled participants, and gender ratios, as well to insufficient sample sizes and inadequate adjustment for confounders. In the present study, we used data from a large Han Chinese population and found that OSA was independently associated with insulin resistance, but not sleep duration. All of the participants were enrolled from our sleep center and most had severe OSA. Thus, OSA may have masked the small effect of sleep duration on insulin resistance.

Interestingly, the risk of insulin resistance in those with OSA and extremely short sleep duration (< 5 h) was comparable to that of primary snorers with the same sleep duration. These observations suggested that OSA combined with extremely short sleep duration had a greater detrimental effect on insulin resistance than OSA alone. Improving extremely short sleep duration may also improve insulin resistance in OSA.

REM sleep accounts for about 25% of the TST and is associated with distinct physiologic alterations. Few previous studies explored the associations of insulin resistance with respiratory events and sleep duration during REM sleep period in OSA. In our study, we preliminary found that REM predominant OSA was associated with insulin resistance to some extent. However, prospective studies with larger sample size are warranted to verify our findings.

The main finding of our study is that OSA is independently associated with insulin resistance rather than short sleep duration. Continuous positive airway pressure (CPAP) is the first-line treatment of OSA and it could improve insulin resistance through abolishing disturbed respiratory events during sleep. Besides, we also found that REM predominant OSA was associated with insulin resistance to some extent. Thus, clinicians should pay more attention to increase CPAP usage time during REM sleep alleviate insulin resistance of OSA.

Despite the large sample size, our study had several limitations. First, as this was a cross-sectional study, it was not possible to determine the causal relationships of short sleep duration, OSA, and insulin resistance. Second, our sample was composed predominantly of male patients, who typically had OSA. This imbalance may have led to bias in the prediction models. Third, as this was a hospital-based study, the generalizability of our conclusions to asymptomatic patients, such as those residing in the community, is unclear. Fourth, though we carefully excluded participants who had medication which could affect sleep or insulin resistance, however, other drugs such as anti-hypertension and lipid-lowering drugs might also potentially influence the reported associations. Fifth, sleep duration was determined based on one overnight PSG session, but night-to-night variability and first-night effects cannot be excluded. In contrast, actigraphy performed using a simple portable can record sleep duration continuously for 7 days. Although PSG was not the optimal choice, good agreement between actigraphy and PSG with respect to the monitoring of sleep duration has been established in OSA [32].

## Conclusion

In conclusion, the results of the present study suggested that OSA, but not short sleep duration, is correlated with insulin resistance. It is worth noting that short and extremely short sleep durations (< 5 h) combined with OSA have a more detrimental effect than OSA alone with regard to insulin resistance. Further prospective cohort studies are warranted to determine the causal relationships between OSA, short sleep duration, and insulin resistance, and to ascertain whether improving sleep time in turn improves insulin resistance in patients with OSA.

## Supplementary information

**Additional file 1.**

## Data Availability

The datasets used and/or analysed during the current study are available from the corresponding author on reasonable request.

## Abbreviations

OSA: Obstructive sleep apnea
PSG: Polysomnography
CVD: Cardiovascular diseases
BMI: Body mass index
NC: Neck circumference
WC: Waist circumference
HC: Hip circumference
WHR: Waist/hip ratio
HOMA-IR: Homeostasis model assessment for insulin resistance
SBP: Systolic blood pressure
DBP: Diastolic blood pressure
TC: Total cholesterol
TG: Triglyceride
HDL-C: High-density lipoprotein cholesterol
LDL-C: Low-density lipoprotein cholesterol
ApoA-I: Apolipoprotein A-I
ApoB: Apolipoprotein B
ApoE: Apolipoprotein E
Lp(a): Lipoprotein (a)
ESS: Epworth Sleepiness Scale
AHI: Apnoea–hypopnea index
ODI: Oxygen desaturation index
MAI: Micro-arousal index
REM: Rapid eye movement
