# Supplementary material for: Interaction between obstructive sleep apnea and short sleep duration on insulin resistance: a large-scale study: OSA, short sleep duration and insulin resistance
Source: Respir Res. 2020 Jun 16;21:151. doi: 10.1186/s12931-020-01416-x (PMC7298870; doi:10.1186/s12931-020-01416-x)
Supplement: Supplementary file 1 — Additional file 1. [file 12931_2020_1416_MOESM1_ESM.docx]

Supplementary Table1

Table 9 adjusted ORs and 95% CIs for the association of insulin resistance and REM sleep predominant OSA

| predictors | OR (95% CI) | | | | |
| --- | --- | --- | --- | --- | --- |
|  | Model 1 | Model 2 | Model 3 | Model 4 | Model 5 |
| Primary snoring | Reference | Reference | Reference | Reference | Reference |
| REM sleep predominant  OSA | 1.355(1.019-1.802)^*^ | 1.328(0.992-1.778) | 1.328(0.992-1.778) | 1.277(0.947-1.721) | 1.177(0.861-1.609) |

Model 1 was adjusted for age, sex and BMI; model 2 was adjusted for variables included in model 1 and smoking, alcohol use, hypertension, hyperlipidemia, time in bed, S3, ESS; model 3 was adjusted for variables included in model 2 and TST; model 4 was adjusted for variables included in model 3 and waist hip ratio; model 5 was adjusted for variables in model 4, diabetes mellitus, apolipoprotein A-1, apolipoprotein-B, apolipoprotein-E and lipoprotein-α.Acronyms: OSA, obstructive sleep apnea; OR, odds ratio; CI, confidence interval; BMI, body mass index; TST, total sleep time; ESS, Epworth Sleepiness Scale; AHI, apnea-hypopnea index.

*p indicates a significant difference.

Supplementary Table2

Table 8 Adjusted odd ratios (ORs) and 95% confidence intervals (CIs) of potential confounding factors for insulin resistance

| predictors | OR (95% CI) | |
| --- | --- | --- |
|  | Model 1 | Model 2 |
| BMI (kg/m^2^) | 1.319(1.290-1.349) | 1.311(1.2811.341-) |
| Hypertension | 1.149(0.990-1.332) | 1.131(0.975-1.312) |
| Diabetes mellitus | 5.978(4.189-8.531) | 5.865(4.113-8.362) |
| Hyperlipidemia | 1.518(1.273-1.811) | 1.486(1.245-1.775) |
| Smoking | 0.895(0.783-1.023) | 0.890(0.778-1.018) |
| Alcohol consumption | 0.747(0.606-0.921) | 0.741(0.601-0.913) |
| Apolipoprotein A-I (g/L) | 0.511(0.361-0.724) | 0.500(0.352-0.708) |
| Apolipoprotein-B (g/L) | 1.781(1.227-2.586) | 1.713(1.177-2.493) |
| Apolipoprotein-E (mg/dL) | 1.196(1.145-1.248) | 1.193(1.143-1.246) |
| Lipoprotein-α (mg/dL) | 0.991(0.986-0.995) | 0.991(0.987-0.995) |

Model 1 was adjusted for BMI, hypertension, diabetes mellitus, hyperlipidemia, smoking, alcohol consumption, apolipoprotein A-1, apolipoprotein-B, apolipoprotein-E and lipoprotein-α; model 2 was adjusted variables in model 1, obstructive sleep apnea and total sleep duration.
